# Advances in Animal Anatomy

**DOI:** 10.3390/ani13061110

**Published:** 2023-03-21

**Authors:** Matilde Lombardero, María del Mar Yllera

**Affiliations:** Department of Anatomy, Animal Production and Clinical Veterinary Sciences, Unit of Anatomy, Faculty of Veterinary Sciences, Campus of Lugo, University of Santiago de Compostela, 27002 Lugo, Spain

This Special Issue was the result of reviewing Leonardo da Vinci's anatomical drawings of the bear foot and the horse trunk (among others) [1]. Since then, we were challenged to propose an interesting topic under the title “Advances in Animal Anatomy”. We were convinced that we had to opt for more than just descriptive anatomy; we chose applied anatomy since, at present, the time dedicated to teaching anatomy has been drastically reduced in the undergraduate programs of veterinary degrees in favor of subjects from the clinical field/area. We also decided to address both domestic and exotic animals, which are so prevalent today in veterinary practice, as well as those animals that must be kept in rehabilitation and conservation centers. Unfortunately, veterinarians are not very familiar with the anatomy of the latter species (which always tends to be considered similar to that of domestic animals), their physiology and more frequent pathologies, since these are not part of the core training in the veterinary degrees. This deficiency is probably not exclusive to the veterinary undergraduate programs. Researchers and specialized technicians who work in wildlife recovery centers and zoos and are involved in exotic medicine and the welfare of these species that are under their surveillance, also need specialized information regarding these species’ anatomies and physiologies.

In order to shed light on the applied anatomy of domestic, exotic, and wild species, we introduce the fourteen manuscripts (eleven research articles and three reviews) that are compiled in this Special Issue (Figure 1), covering current research trends in applied anatomy by using a wide range of techniques. Computed-tomographic imaging was used in four articles, magnetic resonance imaging in three of them, radiography in two articles, and ultrasound techniques and endoscopy in addition to the traditional gross dissections, morphological, and morphometrical studies were also employed (Figure 2).

The horse is the species that most articles, including three research articles and one review, in this Special Issue study. Phalanges from the equine hand were studied by Gündemir et al. [2] by using X-ray imaging. They took radiogrametic measurements of the forelimb phalanges of Arabic and throughoutbred horses. Based on the left manus data, they stated that the proximal and middle phalanges could show sexual dimorphism. These two phalanges can also be used to differentiate breeds, especially, considering the depth of the caput of the proximal phalanx, which can reaching an accuracy level of breed classification of almost 90%. Moreover, all the radiometric measurements made to the thoracic limb phalanges of 75 horses are available as valuable reference data/material when evaluating the manus digital bones in horses.

The second research article about horses focuses on equine dentistry. The most accessible maxillary teeth of the horse, the incisors, were studied by Miró et al. [3] in 25 skulls from horses aged between 12–42 months. Combining visual inspection, radiographic study and computed-tomography imaging, they investigated the development of the deciduous incisors, the dental germs of permanent incisors, and the surrounding bone, just before and up to the beginning of teeth shedding. Measurements of three lengths and two proportions or relative lengths were also analyzed. The development of deciduous and permanent maxillary incisors, in addition to their alveoli, was described in detail at different ages. It was concluded that the dental germ of the first permanent incisors appears at 12 months, although their crown mineralization starts a few months later.

The third manuscript that focuses on horses addresses the study of the thorax in neonatal foals. Authored by Arencibia et al. [4], their study was based on computed-tomography angiography (CTA) complemented with the gross dissections and sectional anatomy of the same specimens used as anatomical references. With intravascular contrast, CTA gives an overview of the thoracic morphology, providing information on the size and position of the heart, as well as the heart chambers, in addition to the information on the main thoracic blood vessels. This information could be used as reference data when comparative morphology is needed to reach a diagnosis in foals with thoracic disease. A dorsal view is preferable to visualize midline thoracic vascular structures (including pulmonary vessels and the brachiocephalic trunk), while lateral views are better to reveal the relationship between the heart chambers and the main blood vessels.

The other species of domestic mammals studied in three of the articles were the dog and the cat. The research article by Ariete et al. [5], which referred to the canine internal vertebral venous plexus (IVVP) of the lumbar segment, used contrast-enhanced computed-tomography (CT) to conduct a morphometrical study of the aforementioned vessels, the dural sac, and the vertebral canal. As part of the vertebral venous plexus, this IVVP is made of a paired thin-walled and valveless venous vessels with a rhomboidal pattern, placed on the floor of the vertebral canal, and included in the lumbar epidural space. It drains blood from the vertebral column, spinal cord and its meninges, including the paravertebral muscles. As a result, when bursting, spontaneous spinal epidural hematomas are produced and could generate spinal cord compression and progressive neurological disorders. IVVP is also supposed to be involved in the pathogenesis of many other pathologies. Consequently, the accurate morphometric study of the lumbar IVVP (and surrounding structures) in healthy and alive animals is necessary to obtain reference values and to, afterwards, assess their alterations in pathological states. The measurements of the cross sectional area of the vertebral canal, dural sac, epidural space, and the right and left IVVP of six dogs were obtained, in addition to the percentage occupied by the IVVP in relation to the vertebral canal and epidural space, and the proportion of the vertebral canal occupied by the dural sac. Regarding all the measurements, there was a turning point between L4 and L5, showing a clear change in the trends at that level, which is in concurrence with the emergence of the nervous roots of the lumbosacral plexus.

Regarding the domestic cat, two review articles assessed its mandible anatomy and its manipulation when a mandibular pathology had to be treated. The first of them, Part I by Lombardero et al. [6], analyses the mandible anatomy in detail in order to obtain a deep knowledge of its morphology and avoid iatrogenic damage, caused by the fact that they are usually treated as those of small dogs. The cat mandible has fewer dental pieces than the mandible of dogs, and most of its mandibular body is filled up with dental roots and the mandibular canal (with the neurovascular supply), leaving little bone surface for the safe placement of screws in a fracture resolution. It should be emphasized that the mandibular canal is not a medullary canal and intramedullary pins should be avoided. The innervation areas of the different branches of the inferior alveolar nerve (with afferences to both skin/mucosal and tooth/periodontal structures) were also considered to serve as a reference when a mental nerve blocking must be performed. In addition, other specific considerations include the angular process in the ventrocaudal part of the mandibular ramus when the mouth remains wide open (with mouth-gags) for a considerable time, which presses on the maxillary artery. This compression reduces its blood flow (mainly directed to the brain since a functional internal carotid artery is missing in cats) producing temporary or permanent neurological disorders due to a cerebral ischaemia. Consequently, in order to prevent this complication, the use of spring-loaded mouth gags to keep their mouth wide open should be avoided in cats. Complementary to this information, Part II, also by Lombardero et al. [7], described different mandibular fractures and temporomandibular joint dislocations and how they could be solved when possible non-invasive techniques should be considered first. Otherwise, it was recommended that simple jaw fractures should be used to repair caudal to rostral, preferably, using a ventral approach. Diverse surgical methods were discussed to maintain biomechanical functionality when repairing mandibular fractures. However, taking into account that the use of rigid fixation methods, such as osteosynthesis plates, are challenging due to the scarce availability of bone surface to fix the screws onto, a new prosthesis design was proposed by the authors to repair simple mandibular body fractures. This prosthesis proposal was a conceptual design to provide an acceptable rigid biomechanical stabilization while minimizing dental root and neurovascular damage, hence reducing patient suffering and speeding up their recovery. This prothesis would be custom-designed and would have to be manufactured in a biocompatible and resistant material, such as titanium. Its shape would look like two horizontal “Y” partially overlapped, and it would support three points of fixation with small screws, each one strategically placed to avoid damaging any tooth, periodontal structure or branch of the inferior alveolar nerve. The fourth fixation point was a flat hook-like flap embracing the body’s ventral border, caudal to the mandibular fracture, thus keeping the integrity of the mandibular canal and its neurovascular supply, thus contributing to the patients’ welfare. Custom-designed prostheses are utterly dependent on reliable technology, from diagnostic imaging (such as virtual surgical planning—VSP—or cone-beam computed tomography—CBCT) to computer-aided design/computer-aided manufacturing (CAD/CAM) technology. All together, they are the current trend and the trend of the near future.

The Bengal Tiger (*Panthera tigris tigris*) belongs to the same family as the domestic cat, *Felidae*, but it is exotic and much bigger. The elbow joints of the Bengal Tiger (*Panthera tigris tigris*) were studied by Encinoso et al. [8] using magnetic resonance imaging (MRI) in combination with traditional gross dissections. The elbow joint is complex and consists of a hinge joint between the humerus (whose distal end is a trochlea) and the proximal ends of the radius and ulna (with a reciprocal shape), as well as the pivot joint between the proximal ends of the antebrachial bones, all enclosed by a single capsule filled with synovial fluid. Unfortunately, not much information is currently available regarding the regional anatomy of the tiger elbow. Thus, gross dissections of the elbow joint, complemented by the brachial and antebrachial muscles and tendons, and their visualization/identification in MRI images would help us understand the normal tiger elbow anatomy, which can help us discern whether there is any elbow joint disorder in this species. The musculoskeletal system is usually studied by MRI, as it avoids ionizing radiation and it provides good image resolution and good contrast, even in soft tissues. In addition to bones, muscles, ligaments, and articular cavities filled with synovial fluid were observed with MRI, and all of these joint structures were confirmed by gross dissection. The study of the tiger elbow joint by means of MRI is essential for the proper training of experts involved in Bengal tiger conservation (such as veterinarians and researchers), as it will allow them to acquire a deep knowledge of the healthy elbow joint, and consequently, to be able to identify any alteration, reaching an accurate diagnosis, treating the patient successfully, minimizing suffering, and promoting animal welfare.

Additionally, the wild relative of the domestic dog, the Iberian wolf, was studied in this Special Issue in a study of their teeth, using a morphological and morphometric approach. This interesting dental analysis, led by Toledo et al. [9], is a comprehensive study of the dental type (incisors, canines, premolars, and molars from maxilla and mandible) morphology and morphometry, including any sexual dimorphism, and was carried out on 45 skulls of Iberian wolves (males and females) with permanent dentition. Up to 36 dental variables (including superficial and deep bite marks) were assessed and statistically analyzed to obtain a valuable morphometric data collection to establish their age and sex in a population control. In addition, the results of the analysis of their bite mark patterns (based on the tooth mark dimensions, distribution, and proportions) could be used as a database reference to differentiate between domestic dogs and Iberian wolves in forensic cases when an identification of a bite mark is needed in cases of livestock attack. This can aid in receiving economic compensation. From here on we will address the topic of mammals that live in the sea. This Special Issue includes three research articles that address the anatomical study of the whole head of dolphin fetuses and newborn dolphins, and the developmental and comparative study of some species of dolphins focused on different regions of the head (oral, pharyngeal and nasal cavities). Regarding García de los Ríos et al.’s [10] the developmental study on the head of striped dolphins during the fetal and perinatal periods, they used different techniques, from gross dissection and sectional anatomy to diagnostic imaging (CT and MRI), to study the anatomical and physiological inferences affecting the heads of marine and land mammals. The development of the head reveals the evolutionary changes of Cetaceans that helped them adapt to the marine environment. These changes include modifications in the feeding apparatus, the caudal relocation of the nasal opening, and other structures that were reduced or even removed. Currently, there are not many methods to estimate the age of dolphin specimens during the fetal period, thus they used CT and MRI in correlation with transversal sections to study the anatomical changes of the heads of the four specimens, which occurred in chronological order during gestation. The results were organized into nine subsections covering oral cavity, rostrum, melon, nasal cavity and paranasal sinuses, orbit and eyeball, central nervous system, ear, larynx, and cranial cavity. This article is richly illustrated with photographs, so they can be used as a head morphology atlas for these species of odontocetes, giving valuable information about the changes produced in the head during prenatal and postnatal development, which are discussed in detail under an anatomical and functional perspective. This general study serves as an introduction to the next two articles, which address different structures of the head in more detail. The second article by García de los Ríos et al. [11] studied the comparative anatomy of the nasal cavity in three species of dolphins, covering the external nose and nasal cavity in different stages of development. They used endoscopy, MRI, anatomic sections and dissections, and histology, as well as the CT to generate 3D reconstructions of the nasal cavity and the bones that delimit it. The external nose is protected by two nasal lips (rostral and caudal) becoming gradually more waterproof during the development to adulthood. Their nasal cavity, usually with vertical orientation, has a single nostril continuous with a vestibule (with two vestibular folds or phonic lips, two diverticula, and two incisive recesses) and two nasal cavities (right and left) at both sides of the nasal septum, conducting the air towards the choanae. Their nasal cavity has no nasal cornettes or conchae, and it is divided in two parts: respiratory and olfactory. However, no olfactory epithelium was identified. All findings were profusely illustrated with 30 figures, each of them composed of a panel of photographs. The latest article by García de los Ríos et al. conducted a developmental study of the oral and pharyngeal cavities [12] of five species of dolphins, also based on endoscopy, MRI, anatomic sections and dissections, and histology. They increased the number of species compared to their previous work on the nasal cavity [11] and studied specimens from a wide range of ages, from fetuses, through to newborns, juveniles, and adults. They described in detail an oral cavity divided in a vestibule and an oral cavity proper, with a roof and a floor, the tongue, and teeth. They reported the pharyngeal cavity divided in three parts and confirmed the existence of the pair of pharyngeal diverticula of the auditory tubes from the early stages of development, with two areas inside: the cavity lined by respiratory epithelium (in contact with air) and its walls with a pharyngeal vascular plexus that would help to eject the air into the adjoining nasopharynx, relevant in decompression. Taken together, these three articles [10,11,12] present valuable descriptive and visual information about the anatomy of the head structures studied in some species of dolphins during development, relevant in veterinary medicine and some fields of biology.

Moving from mammals to reptiles, this Special Issue contains a study of the head of the monitor lizard by Pérez et al. [13], which includes advanced imaging techniques, such as CT and 3D head reconstruction in order to explore the complex anatomy of the head and associated structures of these animals. They used sagittal and transverse CT images, volume-rendered reconstructed CT images, as well as maximum intensity projection (MIP) images to map the head of the Komodo dragon. This is valuable visual work, with all the main structures of the head identified, including the comparative images from CT bone and soft tissue windows at different transversal levels, the 3D volume-rendered reconstruction images from dorsal, ventral, and lateral views, complemented with the dorsal and ventral MIP images, display the head bone structure. Apart from the bones and other structures, including muscles, nervous structures (brain, cerebellum, and brain stem), eye (different structures, as well as sclera ossicles), glands (labial, nasal and Harderian glands), and air-filled structures (oral and nasal cavities, larynx, and trachea), the existence of cephalic osteoderms was also verified with the techniques used. This kind of morphological study based on CT imaging is very useful for people in charge of wildlife conservation centers, such veterinarians, researchers, and technicians, who have to deal with the day-to-day prevention and treatment of threatening diseases in this species in captivity.

Turning to amphibians, and to conclude the list of research articles, Ruiz-Fernández et al. [14], using non-invasive methods, such as benchtop MRI (BT-MRI) and high-resolution ultrasound (HR-US) techniques, managed to identify the biological sex of two species of Anuran in sexually mature specimens when sexual dimorphism is not apparent. BT-MRI is based on expensive equipment and the patients should be under anesthesia. The resulting images were more accurate, allowing for the identification of their sexual organs. Nevertheless, the equipment for HR-US is more affordable for veterinary clinics and zoo facilities, and has some advantages, such as that patients do not need anesthesia and that it is less time consuming. Using it, they identified the gonads of both species. The ovaries were clearly distinguished (as they appeared hyperechoic, with plenty of hypoechoic foci—follicles); however, the testes were not so evident due to their homogeneous echotexture. Therefore, amphibian sexing using non-invasive techniques is a significant advance in the conservation of these species and in reproduction programs for them.

To conclude the editorial of this Special Issue, we return to the initial article about Leonardo da Vinci’s drawings representing the anatomy of the foot of the bear and the horse [1]. The bear foot was considered one of his early scientific drawings and, apart from being a very common animal in Italian mountains, he probably chose to dissect the bear foot because of its plantigrade gait. Later on, his masterpieces on human anatomy were based on his lifelong learning. He was an outstanding Renaissance scientist, as he expressed throughout his life a continuous interest in learning and exploring the world around him. He also did not hesitate to go a little further. This is the purpose of this Special Issue as well, moving forward, displaying the advances of applied anatomy. We do hope to have achieved this and we wish that we all maintain our interest in broadening our knowledge in applied animal anatomy, parallel to the advances in diagnostic technology.

## Figures and Tables

**Figure 1:**
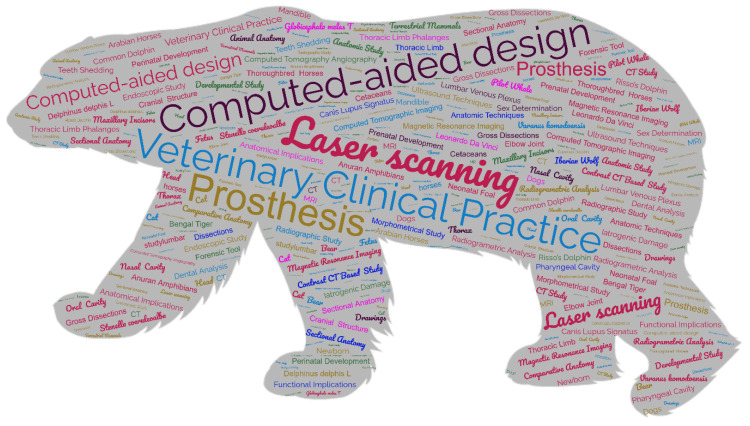
A tag cloud including the titles and keywords of the articles published in this Special Issue.

**Figure 2:**
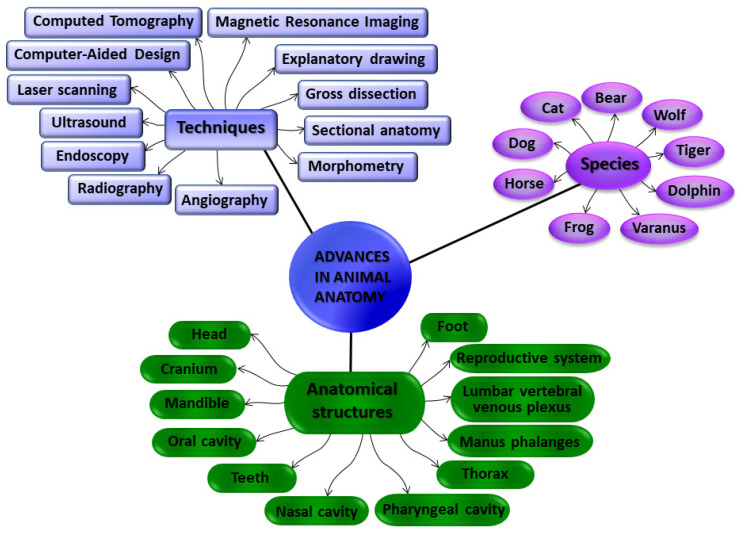
A mind map of this Special Issue summarizing the techniques used to study the different anatomic structures from the diverse species.
